# New strategy for sepsis: Targeting a key role of platelet-neutrophil interaction

**DOI:** 10.4103/2321-3868.135487

**Published:** 2014-07-28

**Authors:** Xu Wang, Weiting Qin, Bingwei Sun

**Affiliations:** Department of Burns and Plastic Surgery, Affiliated Hospital, Jiangsu University, Zhenjiang, 212001, Jiangsu, China

**Keywords:** Sepsis, neutrophil, platelet, interaction

## Abstract

Neutrophil and platelet are essential arms of the innate immune response. In sepsis, platelet abnormal activation as well as neutrophil paralysis are well recognized. For platelet, it is characterized by the contribution to disseminated intravascular coagulation (DIC) and the enhanced inflammation response. In terms of neutrophil, its dysfunction is manifested by the impaired recruitment and migration to the infectious foci, abnormal sequestration in the remote organs, and the delayed clearance. More recently, it has been apparent that together platelet-neutrophil interaction can induce a faster and harder response during sepsis. This article focuses on the activation of platelet, dysfunction of neutrophil, and the interaction between them during sepsis and profiles some of the molecular mechanisms and outcomes in these cellular dialogues, providing a novel strategy for treatment of sepsis.

## Introduction

Sepsis is a combination of clinical manifestations of systemic inflammation specifically related to an infectious insult[1] and the inflammatory dynamic of it, in term of the current hypothesis, includes an initial systemic inflammatory response syndrome (SIRS) followed temporally by a compensatory anti-inflammatory response syndrome (CARS)[2–4] then with a continuously, highly mixed anti-inflammtory response syndrome (MARS).[5] Given a profoundly impairment and life threatening of sepsis, there is an imperative to understand the concrete pathophysiology during sepsis and over the time the understanding is evolving. Mortality from sepsis continues to be high. Totally, the mechanism of sepsis is complex and the late therapies targeting a single molecular fail to cure the disease, for example, the monoclonal antibodies against tumor necrosis factor (TNF)-α,[6] the receptor antagonists of interleukin (IL)-1β[7] and the antibodies to endotoxin.[8] Hence, understanding the intricate and heterogeneous of sepsis addresses a better approach the problem of sepsis. Besides shock and multi-organ dysfunction occurring following the intense inflammatory reaction to sepsis, complications arising from sepsis-related platelet activation and platelet-neutrophil interaction contribute to the morbidity and mortality from sepsis. This review explores the basis for sepsis-related platelet activation, neutrophil dysfunction, and platelet-neutrophil interaction and discusses their clinical implications for the treating intensivist.

**Table:** 

Access this article online	
**Quick Response Code**:	**Website**: www.burnstrauma.com
	**DOI**: 10.4103/2321-3868.135487

## Sepsis-induced platelet activation

Platelets, small (approximately 3–5 µm) anucleate cells derived from bone marrow megakaryocytes, were primitively recognized to sense damaged vessel endothelium and accumulate at the site of the vessel injury to initiate blood clotting. Recently, there is increasing evidence suggesting their indispensable role in regulating inflammatory response.[9–11] During sepsis, platelets are immoderately activated by various pathogen-associated molecular patterns (PAMPs) and damage-associated molecular patterns (DAMPs), which amplify inflammatory response through complicated mechanisms. And the response triggered by the interaction between platelets and various PAMPs and DAMPs is through the platelet receptors, mainly glycoprotein (GP)IIb-IIIa (mediating the crosslinking of platelet by fibrinogen to promote aggregation),[12] GPIbα (inducing platelet activation mainly by the von Willebrand factor [vWF]), FcγRIIa (enhancing the function of GPIIb-IIIa and GPIbα in an lgG-independent manner),[13,14] complement receptors (increasing upon activation,[15] and inducing platelet aggregation in a complement-dependent process[16]) and toll-like receptors (TLRs). Of note, GPIIb-IIIa, GPIbα, and FcγRIIa play a crucial role in platelet activation, adhesion and aggregation. For TLRs, especially TLR4 and TLR2, they can activate platelet to release immunomodulatory agents (like TNF-α[17]) and promote other cells activation, such as neutrophils, endothelial cells. Inappropriate activated platelets are major contributors to the initiation of disseminated intravascular coagulation (DIC) that is initiated by tissue factor (TF), leading to the platelet adhesion induced by the receptors (like p-selectin) and ligands, like P-selectin glycoprotein ligand (PSGL)-1, interaction,[18] the formation of thrombin, fibrin, and intravascular thrombi,[19] which reduce oxygen supplement and enhance inflammatory cytokine networks.[20,21] Additionally, various pro-inflammatory factors in platelets granules are released into the surrounding environment or transferred to plasma membrane, such as interleukins, monocyte chemoattactant protein (MCP)-1, platelet factor (PF)-4, to activate more remote platelets and immune cells.[22–24] Activated platelets can also release some microparticles.[25] Circulation microparticles are membrane-derived nano-fragments (0.05-1 µm) which contain a storage pool of TF and express P-selectin and platelet glycoprotein IIb-IIIa. As described above, these bioactive molecules may play deleterious role in the dissemination of coagulopathy and inflammatory responses in sepsis.[26]

## Neutrophil dysfunction in sepsis

Neutrophil originates from the bone marrow with a consequent egress to the blood, recruits and migrates to the inflammatory site, then culminates in clearance. The life of neutrophil has been described, all of which are uncontrolled altered during sepsis.

The mature neutrophil within the bone marrow can rapidly egress in the early phrase of sepsis, increasing the circulation numbers by tenfold within a matter of hours compared with the normal condition.[27] The release of neutrophils from bone marrow to the infection site has been historically attributed to the chemotactic factors including leukotriene B4, C5a, chemokine interleukin IL-8,[27–30] and the bacterial products. And the chemokine (C-X-C motif) ligand (CXCL)12, a recently new and pivotal chemoattractant serve to retain neutrophil within the marrow, are also involved in this process.[31–34] The granulocyte colony-stimulating factor (G-CSF) which is indirectly mobilized neutrophil through shifting the balance between stromal cell-derived factor (SDF)-1 and CXCR2 ligands in bone marrow[35] are also associated with this phenomenon, triggering a release of neutrophil into the circulation.

Infection is an alarming condition that renders host to defend. Neutrophil, as the fist line cell against the bacterial and fungal pathogens, recruits to the site of infection. The classical leukocyte recruitment cascade involves the following recognized steps: Tethering, rolling, adhesion, crawling, and, finally, transmigration. This process is a sequential, multistep adhesion cascade in which various cytomembrane molecules are sophisticated interactived (reviewed in Table 1[36]). However, during sepsis, this response is dysregulated with the abnormal accumulation of neutrophil, impaired recruitment of neutrophils to the infectious foci, and damaged neutrophil migration. Of note, the neutrophil cell membrane altered, becoming more rigid and less deformed, and this change in rigidity increases proportionally with sepsis severity.[37] As a result, neutrophils sequester in the capillary beds, especially those in lung and liver sinusoids and the process will lead to microvascular occlusion, resulting in the tissue ischemia and subsequently multiple organ failure.[37,38] Nitric oxide (NO) and its producer inducible nitric oxide synthase (iNOS), the *sine qua non* in neutrophil migration impairment, downregulate neutrophil migration mainly from the following three aspects:

1. The iNOS inhibits leukocyte β-integrins and selectins as well as downregulates vascular cell adhesion molecule (VCAM)-1[39,40] and;
2. NO interacts with other molecules like reaction oxygen species (ROS), forming peroxynitrite that can decrease neutrophil chemotactic activity[41] and leukocyteendothelium interaction which relays on P-selectin[42,43];
3. NO can induce heme oxygenase (HO)-1 expression, one that can impair neutrophil rolling and adhesion.[44,45]

**Table 1:** **Adhesion molecules involved in different stages of the classical neutrophil migration cascades in postcapillary venules**

Different stages	Molecules on endothelium	Molecules on neutrophil
Tethering and rolling	P-selectin	PSGL1 (positively regulates recruitment) PTX3 (negatively regulates recruitment)
Slow rolling	ICAM1	LFA1 (PSGL1-induced)
	E-selectin	PSGL1, ESL1, CD44
Arrest and adhesion	ICAM1	LFA1
	VCAM1	VLA4
Crawling	ICAM1	MAC1
Transmigration and diapedesis	ICAM1, ICAM2	LFA1, MAC1
	VCAM1	VLA4
	CD99	CD99
	PECAM1	PECAM1
	JAMA	LFA1, JAMA?
	JAMB	VLA4
	JAMC	MAC1
	CD99L2	?
	VE-cadherin (negatively regulates recruitment), ESAM	Between endothelial cells

CD99L2 = CD99 antigen-like protein 2, ESAM = Endothelial cell-selective adhesion molecule, ESL1 = E-selectin ligand 1 (also known as GLG1), ICAM = Intercellular adhesion molecule, JAM = Junctional adhesion molecule, LFA1 = Lymphocyte function-associated antigen 1, PECAM1 = Platelet/endothelial cell adhesion molecule 1, PSGL1 = P selectin glycoprotein ligand 1, VCAM1 = Vascular cell adhesion protein 1, VE cadherin = Vascular endothelial cadherin, VLA4 = Very late antigen 4[36]

Besides, the enhanced level of carbon monoxide (CO) and bilirubin in serum and exhaled breath of septic patients[46,47] also indicate that HO-1 pathway plays a role in this pathology. The proteins on the cell-surface and the nuclear as the receptor like C-X-C chemokine receptor (CXCR) type 2, C-C chemokine receptor (CCR) type 2, and peroxisome proliferator-activated receptor (PPAR)γ, mediate the impairment in neutrophil migration. Their participation can be justified by:

1. Decreased expression of CXCR2 on neutrophil isolated from septic patients[48] and the upregulation of CCR2 on circulating murine netrophil during sepsis[49] have been found, which are due, at least in part, to the TLR signaling[50–52];
2. The expression of PPARγ increased in the isolated neutrophil from not only septic mice but septic patients.[53]

Another novel finding is that the direction of neutrophil migration was error during sepsis[54] and the consequence is complex *in vivo.* The precise mechanism about how neutrophils direct to the target destination is incompletely understood.

To maintain the homeostasis of neutrophils, the key thing is a fine management of the balance between the income and outcome neutrophils. Homeostatic removal of neutrophils mainly gives the credit to the macrophages[55] and to a small extent by the dendritic cells and lymph nodes. In it neutrophil undergoing apoptosis allows removal by scavenger macrophage[56] and constitutive apoptosis of neutrophil is an essential factor for keeping neutrophil homeostasis. However, in patients with sepsis the apoptosis of neutrophil is delayed[57–59], which may contribute to tissue injury associated with the multiple organ dysfunction syndrome (MODS) of sepsis. The mechanisms that govern this process are not completely understood and the recent investigation found that the inflammation mediators, i.e., granulocyte-macrophage colony-stimulating factor (GM-CSF), IL-18[60,61], which regulate the pro- and anti-apoptosis genes leading to the change of apoptosis relevant factors expression: B-cell lymphoma (BCL)-2 members,[62] the sFas, Dad1,[63] etc., can manage it. Remarkably, additional upstream regulatory factors of these apoptosis factors are involved in the delayed apoptosis of neutrophil in sepsis. In addition the destructed mitochondrial transmembrane potential and the reduced activity of caspase 3,9[59] also dampen the apoptosis. Along with the death combined with a formation of neutrophil extracellular traps (NETs) which contain nuclear components (like deoxyribonucleic acid, DNA and histones) are decorated by various proteins.[64] During sepsis, NETs present like a double-edged swords: They can trap microorganisms [64] through NET-localized molecules; moreover, they exert detrimental effects that contribute to tissue damage.[65]

### Platelet-neutrophil interaction during sepsis

Platelets and neutrophils have the potential to promote inflammatory response during sepsis independently of each other, but together platelet-neutrophil interactions can induce a faster and harder response.[11,65] In the early phase of sepsis, possibility of collisions between platelets and leukocytes is promoted by the rheological margination of neutrophil exiting the central core of the blood vessel. With further activation by septic inflammatory stimuli (PAMPs and DAMPs), platelet-neutrophil interactions are extensively formed.[66] It is well accepted that activated platelets adhere to neutrophils through a rapid surface expression of a granular protein P-selectin that binding to the high affinity counter ligand PSGL-1 expressed on neutrophils.[67–69] Engagement of PSGL-1 leads to further neutrophil activation of the β_2_-integrins, CD11a/CD18, LFA-1 (αLβ_2_), CD11b/CD18 and Mac-1 (αMβ2) that do not require additional stimuli,[70–72] which result in massive neutrophil migration and accumulation in distal organs such as lung and liver to cause tissue injury. Related to this, Clark *et al.* found that isolated human neutrophils require 2–4 hours stimulation to release NET, however it took a few minutes when interact with lipopolysaccharide (LPS)-stimulated platelets under flow.[65] Further studies discover that platelet-induced NET release is dependent on lymphocyte function-associated antigen (LFA)-1 interaction both in murine and human sepsis.[73] Although NET formation is critical for ensnare bacteria, it can also provide a stimulus and scaffold for thrombus formation, by promoting platelet and RBC adhesion and by concentrating effector proteins and coagulation factors involved in clotting to aggravate DIC and tissue damage during sepsis.[73,74]

**Figure 1:**
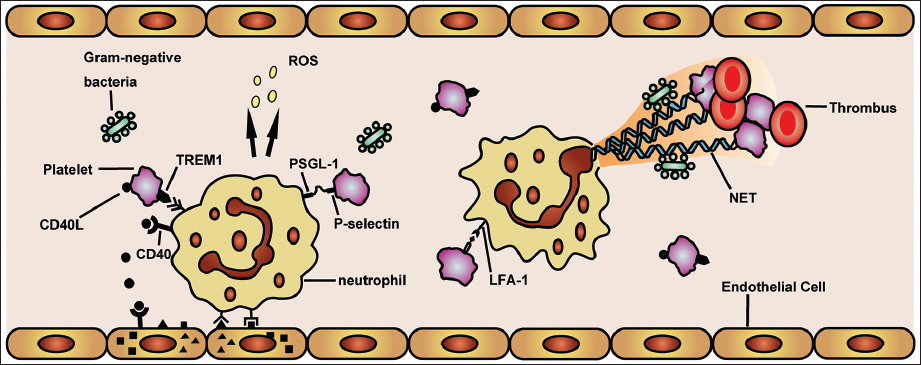
Platelet-neutrophil interaction during sepsis. During sepsis, activated platelets attach to neutrophils via a selectin dependent process, namely the release and expression P-selectin of platelet from a-granules which binds to the counter ligand P-selectin glycoprotein ligand (PSGL) expressed on neutophils. Besides that, activated platelet can expression CD40L and then shed it into circulation. Triggering receptor expressed on myeloid cells (TREM)1, triggering receptor expressed on myeloid cells together with CD40L interact with neutrophils which can further promote the activation of neutophils and its generation of reaction oxygen species (ROS). For platelet-expressed CD40L, it can also interact with CD40 on endothelial cells to stimulate the endothelial cell to a pro-inflammatory phenotype: upregulation of intercellular adhesion molecule (ICAM)1 and vascular cell adhesion molecule (VCAM)1, thereby driving neutrophil recruitment. The platelet can also mediate the formation of neutrophil extracellular trap (NET) via the interaction of lymphocyte function-associated antigen (LFA)-1, which can trap free bacteria and enhance the platelet and red blood cell (RBC) adhesion to promote thrombus formation.

The interaction between CD40 and its ligand CD40L activates various pathways in immune and non-immune cells related to inflammation and was shown to be critical for the development of sepsis.[75,76] Activated by septic stimulation, expression of CD40L is severely increased on platelet surface and shed into circulation to interact with immune cells.[75,77] Platelet-derived CD40L can be sensed by CD40 on endothelial cell to induce upregulation of intercellular Adhesion Molecule (ICAM)1 and VCAM1 and release of CCL2, thereby indirectly promoting leukocyte recruitment to inflammatory sites.[78] In addition, platelet-derived CD40L can directly interact with neutrophil CD40 and enhance the neutrophil activation and ROS generation.[79] Another way in which platelets interact with neutrophils during sepsis is through triggering receptor expressed on myeloid cells (TREM)1.[80] In the presence of LPS neutrophils and platelets interact through TREM1 activation, which increases neutrophil-mediated production of ROS and secretion of IL-8.[81] TREM-like transcript (TLT)1, an orphan receptor only expressed in the α-granules of platelets and megakaryocytes, is newly demonstrated to be significantly upregulated in the plasma of patients with sepsis and correlated with the outcome in these patients.[82] These observations suggest that TREM1 ligand TREM-like family may have synergetic effects on interaction of neutrophils and platelets during sepsis[9] [Figure 1].

Increasing evidences have proved that the interactions between platelet and neutrophil play a major role in the development of organ failure both in septic patients and experimental animals. In patients with sepsis, enhanced platelet-neutrophil interaction was determined by increased platelet-leukocyte conjugates in blood using a double-labeling flow cytometry technique and this interaction correlated with the severity of septic organ dysfunction.[83] Platelets mediate excessive neutrophil recruitment in lung and acute lung injury via CD40L/Mac-1 pathway in murine abdominal sepsis.[79] Neutrophil-dependent recruitment of platelets in the liver microcirculation impairs sinusoidal perfusion and may contribute to the liver dysfunction in murine abdominal sepsis.[84]

### Therapeutic potential for platelet-neutrophil interaction in sepsis

Clinical therapeutic strategy for platelet or neutrophil alone has been applied for several decades and achieved a great success. However, therapeutic strategy targeting platelet-neutrophil interaction in sepsis is barely seen. Come a long way in understanding of molecular and cellular basis of platelet-neutrophil interaction in sepsis, a growing body of studies focuses on the interference with platelet-neutrophil interaction in sepsis. Ogura H *et al.* reported P-selectin-dependent platelet-neutrophil interaction is involved in the outcome of severely septic patients and P-selectin blockade markedly inhibited this interaction.[85] Exposed to cecal ligation and puncture (CLP), CD40L gene-deficient mice show a significantly inhibited platelet-neutrophil interaction and alleviated pulmonary damage.[79] Experimental inhibition of PSGL-1 significantly abolished CLP-induced platelet-neutrophil aggregation which has no effect on neutrophil expression of Mac-1.[10] Owing to crucial role on platelet-neutrophil interaction, TREM1-silenced mice are highly resistant to a lethal endotoxin challenge and partial silencing of TREM1 in the bacterial peritonitis model produces a significant survival benefit.[86] Exciting times lies ahead, with the improving awareness of intracellular machinery, we are on the cusp of converting new lessons from intravital studies into novel effective treatment options.

## Conclusion

Sepsis, frequently occurs after hemorrhage, trauma, burn, or abdominal surgery, remains a major challenge both for clinicians and researchers. Despite many years of intensive research and numerous clinical studies, its pathophysiology is still incompletely understood, and some specific treatments have not been successful in clinical trials. This is mainly due to the fact that sepsis can be characterized as a complex and dynamic disease process. Targeting platelet-neutrophil interaction is a promising field for sepsis management and infection control. Developing sepsis-specific platelet-neutrophil interaction for patients is a path strewn with obstacles, but an exciting and promising area of research. Understanding of sepsis-induced platelet-neutrophil interaction offers vast opportunities for improving the mortality and morbidity from sepsis. We expect that this novel strategy will continue to be clinically assessed and potentially exploited for the more effective future treatment of sepsis.
